# Economic Evaluation of Environmental Interventions: Reflections on Methodological Challenges and Developments

**DOI:** 10.3390/ijerph15112459

**Published:** 2018-11-05

**Authors:** Laura Bojke, Laetitia Schmitt, James Lomas, Gerry Richardson, Helen Weatherly

**Affiliations:** Centre for Health Economics, University of York, York YO10 5DD, UK; laetitia.schmitt@york.ac.uk (L.S.); james.lomas@york.ac.uk (J.L.); gerry.richardson@york.ac.uk (G.R.); helen.weatherly@york.ac.uk (H.W.)

**Keywords:** economic evaluation, cost-effectiveness, public health

## Abstract

Evaluation of the costs and outcomes associated with environmental policies and interventions is often required to inform public policy and allocate scarce resources. Methods to conduct assessments of cost-effectiveness have been developed in the context of pharmaceuticals, but have more recently been applied in public health, diagnostics, and other more complex interventions. The suitability of existing economic evaluation methodology has been explored in many contexts, however, this is yet to be undertaken for interventions and policies pertaining to the natural environment, such as urban green spaces and strategies to reduce indoor and outdoor air pollution. To make significant inroads into the evaluation of interventions and policies relating to the natural environment requires an understanding of the challenges faced in this context. Many of these challenges may be practical (data-related), however, a number are also methodological, and thus have implications for the appropriate framework for economic evaluation. This paper considers some of the challenges faced when conducting cost-effectiveness analyses in this context and explores what solutions have been proposed thus far. The intention is to help pave the way for consideration of which existing framework is most appropriate for the evaluation of natural environment (NE) interventions, or if a distinct framework is required. Environmental policies and interventions relating to the built environment, for example, housing, are not explicitly included here.

## 1. Background

Many decision-making bodies use economic evidence when deciding which projects to adopt [1]. Economic evaluations incorporate information on the costs and outcomes for each of the competing options that the decision-maker may choose from when allocating scarce resources. Such evaluations can be based on a single study or can synthesise evidence from multiple sources, for example, using a decision analytic model [2].

In health, methods for economic evaluation were established in pharmacoeconomics, but have now been transferred to the appraisal of diagnostics, medical devices, public health interventions, and social care interventions. Across the world, health technology assessment (HTA) agencies have developed guidelines for the appraisal of health technologies. These can be used by organisations as they produce evidence to support reimbursement and pricing for their products, as well as by those considering the credibility and quality of the evidence submitted [3]. Examples include the Guide to the Methods of Technology Appraisal by the National Institute for Health and Care Excellence (NICE) and the Medical Research Councils (MRC) complex interventions guide [4]. There is also more generic guidance relating to the conduct of single study (usually trial-based) [5] and model-based economic evaluations [6].

**Figure 1:**
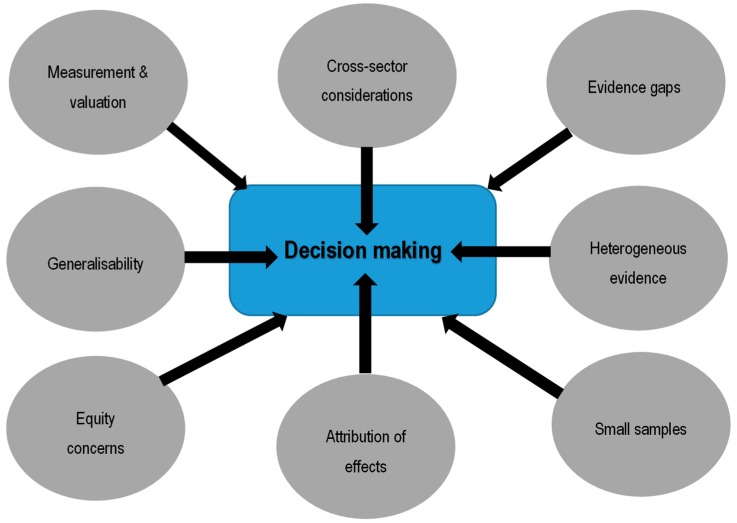
Schematic of challenges in the evaluation of public health interventions.

The many challenges associated with evaluating technologies increase when moving away from the rather more data rich field of medicines to public health [7], where, typically, cost-effectiveness analysis is implemented using quality-adjusted life years (QALYs) including health-related quality of life weights (HRQol) based on social values. The challenges are expected to be further exacerbated when evaluating public health interventions mediated by an improvement in the natural outdoor environment. This may range from the provision or improved access to, green and blue spaces, to the reduction of outdoor environment hazards such as outdoor air pollution or extreme weather events such as flooding, heatwaves, and so on. The challenges in public health have been discussed previously [7] and pertain to the attribution of effects; measurement and valuation of outcomes; broader cross-sectoral costs and consequences; and, increasingly, the requirement to incorporate equity considerations within evaluations (see Figure 1—grey ovals denote challenges when evaluating in public health contexts). Public health evaluation is expanding, including the evaluation of the effectiveness and cost-effectiveness of interventions and policies relating to the natural environment (NE), at international, national, and local levels. As well as health effects, as commonly captured in a pharmacoeconomic evaluation, in an evaluation of a NE intervention, it may be relevant to capture other non-health effects, and in fact these may be the primary objective of the intervention, for example, an improvement in urban walkability or green spaces provision to improve tourism or boost property values.

To make significant inroads into the evaluation of interventions and policies relating to the NE requires an understanding of the challenges faced in this context. These are usefully informed by experts within the field that the evaluation aims to inform as part of a cross-disciplinary team. Many of these challenges may be practical (data-related), however, a number are also methodological, and thus have implications for the appropriate framework for evaluation. This paper outlines the methodological challenges faced in evaluating NE interventions and highlights some of the potential solutions to these challenges and areas for further research. The intention is to help pave the way for consideration of which existing framework is most appropriate for the evaluation of NE interventions, or if a distinct framework is needed.

## 2. Methodological Challenges in Conducting Economic Evaluations of NE Interventions

A number of key methods challenges exist in evaluating NE interventions that impact on (not exclusively) health. Although these are often interrelated, different elements can be described that are not all in accordance with the challenges in public health (Figure 1).

### 2.1. Attribution of Effects

Foremost is to be able to identify whether an NE intervention works. In economic evaluation, this begins with comparing the effectiveness of an intervention relative to the next best alternative. The aim is to demonstrate a causal effect, for example, an outdoor park enhances health, as opposed to simply a measure of association of effect, such as people who live near an outdoor park are healthier than people who do not.

Evidence of causality is best addressed via randomised controlled trials (RCT) [8]. Controlled experimentation is rarely feasible in the economic evaluation of NE interventions because of the cost and complexity of such studies. Evaluations of NE interventions are thus overwhelmingly based on observational data and any causal inference is limited by potential bias arising from uncontrolled baseline differences between intervention and control groups [9]. When considering utilising a before and after approach, it is important to note that NE interventions are often added into a system over time, and so it is difficult to determine when the intervention period begins and the control period stops.

#### 2.1.1. Defining Intervention (Exposure) and Control Groups

Key to identifying intervention and control groups is the notion of exposure to the intervention, which, in the case of NE interventions, is not necessarily straightforward to define and is typically not binary, that is, exposed or not, as would be the case when evaluating a drug.

In the case of interventions that modify the physical environment such as urban green spaces (UGS), proximity to the intervention has been used frequently [10]. However, the cut-off distance after which individuals can be deemed “not exposed” is not necessarily clear, and because one may expect a reduction in intervention effect as the distance from UGS increases, the intervention group will not have a “homogenous exposure”. While graded measures of exposure using distance can be used [11] to distinguish intervention from control groups—in a similar way to the evaluation of differences in effect for a standardised unit difference in air pollution exposure—this approach would still assume that proximity equates with real exposure.

Exposure misclassification thus represents a key challenge to be addressed in evaluations of environmental interventions. As this issue is expected to be non-differential (i.e., to affect both control and intervention groups similarly), it should not lead to bias, but is expected to lead to a loss of statistical power that further complicates the task of attribution of effects.

#### 2.1.2. Non-Linearity in Effects and Uncertain Dose-Response Function

Many of the health-related benefits derived from nature-based outdoors interventions aimed at reducing environmental hazards such as air pollution, flooding, and heatwaves stem from the provision of services by natural ecosystems [12]. For example, green infrastructure such as trees and wetlands have been proposed to absorb air pollution [13] and reduce flooding risk [14]. However, typically, the quantity of ecosystems services is not a linear function of the amount of natural ecosystems structure [15], and, for instance, the amount of air pollutants captured or water retained may not be a simple linear function of the number of trees planted. 

Non-linearity is thus a key issue. However, it can be readily addressed in NE interventions. This is because the evidence on risks due to exposure to a particular factor will accumulate over time. For example, a key component of an economic evaluation of an intervention that reduces the human exposure to particulate matter will need to consider the literature concerning the ‘dose-response’ relationships that have been estimated [16].

In order to most efficiently allocate resources across nature-based outdoors interventions aimed at reducing environmental hazards, economic evaluations will need to specifically account for non-linearity in effects and uncertainty in dose-response functions that will lead to uncertain increasing or decreasing returns to scale.

#### 2.1.3. Effects Scope and Time-Horizon

Environmental interventions are multifaceted and are expected to impact health via several pathways such as by reducing exposure to environmental risk factors, promoting physical activity, and improving mental health [9]. The links between risk factors and health conditions may be inter-dependent and not independently additive. Also, there may be uncertain lags between changes in environmental risk factors and changes in health. For instance, despite extensive epidemiological research, there is still no consensus on the nature of the time lag between pollution concentration reduction and improved health outcomes.

Importantly, the range of effects is expected to be time-scale and context-specific [17]. Flood risk management policies perfectly illustrate the context and time-scale dependence of the range of expected health benefits of reducing environmental hazards. In the short-run, the main health impacts of floods consist of deaths from drowning, injuries, and waterborne diseases [18]. Over a longer time horizon, floods and their damaging economic consequences have often been associated with adverse effects on mental health [19]. There has been heterogeneous reporting of these longer-term health impacts according to the level of socio-economic development within a country, with a tendency to ignore these effects in poorer countries, despite the fact that the effects are likely to be exacerbated in more vulnerable and poorer settings [17].

More generally, any environmental intervention expected to help mitigate risks to human health that are expected to be exacerbated in the future as a result of climate change, such as weather-related extreme events, should be evaluated over a time horizon that is long enough to encompass these impacts. This is the case, for instance, with urban green space that can mitigate urban heat effects [20] and wetland creation/restoration that can help reduce the risk of flooding [14,21]. Consideration of the appropriate time horizon is relevant in terms of both benefits and costs associated with the interventions. There is, however, no consensus about the appropriate discounting factor when evaluating interventions with consequences in the far-reaching future, as exemplified with the heated debate surrounding the choice of discount rate to evaluate climate change impacts in the Stern review [22].

### 2.2. Measuring and Valuing Outcomes

For NE interventions, it might be appropriate to use a broader measure of quality of life or wellbeing, compared with interventions focused solely on health, with an associated health impact (health related quality of life (HRQoL)). The use of the quality-adjusted life year (QALY) is advocated for the appraisal of many health care interventions [23] and there are assessments of the expenditure required to produce an additional QALY for the health care sector, which can be used to express QALYs in terms of health care resources [24]. With different levels of funding, activity, and efficiency across sectors, however, the opportunity cost of resources may well differ even if outcomes are reflected in a common measure. Environmental interventions tend to generate non-health and health outcomes, and to date, monetization is typically used to aggregate these outcomes (in the form of cost benefit analysis, CBA); however, there is great heterogeneity in willingness to pay (WTP) values generated [25]. In addition, decision-makers in public policy may not want to base monetization on individual preferences alone (WTP), and may wish to incorporate information on the ability to meet socially agreed objectives given constrained resources.

### 2.3. Cross-Sector Considerations

The cross sector nature of NE interventions is a relevant methodological consideration. In a health care setting, if the objective is to maximise health outcomes, maximising QALYs subject to the health sector budget constraint makes sense. In contrast, public sector decision-makers for NE interventions are unlikely to be concerned solely with health outcomes, but rather with the consequences across a range of sectors, in terms of resource use and outcomes for various socially valuable objectives. Here, an intervention will be considered to be cost-effective if its benefits are greater than the forgone benefits of displaced interventions. 

Non-health outcomes may include educational attainment and work productivity, and these can impact on different sectors within the economy, as noted above. An evaluation would need to compare any incremental benefits with incremental opportunity costs across these sectors, thus taking a broader perspective. For example, an NE intervention could potentially improve HRQoL, but reduce wellbeing. If the analyst is faced with such a situation, in order to reach a decision about competing interventions, it is necessary to make an assessment of whether, overall, the intervention is beneficial.

Environmental interventions may involve spillover effects beyond the individuals who are directly targeted by the programme, for instance, health impacts on family and networks of friends. Also, there may be costs to individuals, for instance, out-of-pocket costs. Although it is not unique to NE interventions, this does imply additional complexities. In particular, compliance with interventions may be affected by private costs, may influence incentives, and may also have equity considerations.

### 2.4. Equity Considerations

Increasingly, decision-makers require that economic evaluations should explore the equity implications of any decisions regarding reimbursement. In environmental research, under the principle of environmental justice [26], environmental interventions that reduce exposure to health risks should aim towards a fairer distribution of the environmental risk across population subgroups. Under this principle, some interventions may be required to target high risk groups (e.g., individuals living in air pollution hotspots, or the most vulnerable individuals such as the elderly) and doing so may introduce efficiency/equity trade-offs [27]. Some UGS initiatives can also introduce inequalities, by increasing property values in surrounding neighborhoods and thereby pushing out the existing residents who were the original target beneficiaries of the initiatives [28].

In studies that use the QALY, the normal assumption is that the value of a QALY is the same no matter who receives it; therefore, there are underlying equity implications associated with such approaches but, until recently, there has been little focus on this. However, the distribution of QALY gains between population sub-groups is important in tackling inequalities in health or other outcomes [29], and this may be a key objective in some environmental interventions. There may be sub-groups who differ in their access to the benefits of the intervention and in their capacity to benefit from the intervention, which could undermine their opportunity to benefit from such intervention. For example, UGS have been shown to contribute to health inequalities [30].

## 3. Methodological Developments to Address Challenges in the Evaluation of NE Interventions

A number of methodological complexities in the evaluation of NE interventions have been identified and described. While the totality of these complexities may be large, there have been significant efforts towards overcoming methodological challenges. There is also a better understanding about the research that is needed to overcome the remaining challenges. A number of these activities are discussed below.

### 3.1. Attribution of Effects

As discussed in Section 2.1, attributing an effect to an NE intervention is fraught with difficulties, as the latter are, by their very nature, often unamenable to experimental design. However, NE interventions, such as green spaces creation, urban greenways, and low emission zones, for instance, do often provide a “natural” variation in exposure to a health-promoting (or damaging) outdoor environment that can resemble a quasi-experiment, in that the mechanisms determining exposure allocation can be reasonably expected to be independent of the outcome evaluated. These natural experiments can thus mimic random assignment and be exploited to “pave the way” for causal inference, provided enough data can be collected to control for differences between those ‘naturally’ assigned to the intervention and those which are not. Interventions defined as natural experiments can then be analyzed using a combination of techniques including differences in differences, propensity score analysis, and regression discontinuity, as advocated by the recent MRC guidance for evaluating population health interventions [31].

Depending on the nature of the data available pertaining to health outcomes, it may be preferable to focus on the linkage between intervention implementation and change in risk exposure and to link this information with validated “cost-of–illness” secondary data that quantifies the adverse health impact that the intervention is contributing to reducing. For instance, an evaluation of green space creation may try to only estimate how much a green space creation will increase physical activity, and link this result with estimates of the costs of physical inactivity. This is the approach that has been used to date in economic evaluations of urban green space [30].

It is worth noting that the causal findings from natural experiment analysis may be sensitive to the conceptual and methodological choices used in characterizing the boundaries of exposure, be it dichotomous or graded exposure, and in defining the unit of exposure (e.g., area-level estimated versus individuals-based estimates of exposure) [11]. Sensitivity analyses of results to these choices thus seems warranted. 

A decision-analytic model can provide a useful framework to link intermediate or short-term outcomes to longer-term changes in health and to combine evidence from multiple sources to evaluate the interventions over an appropriate timeframe. Such a model can also have value in transferring findings from real populations or sub-groups and to assess population health at the national and local level. In using such techniques, assumptions have to be made to extrapolate evidence over a longer term and it is important to characterise any uncertainties and reflect these in estimates of cost-effectiveness [32].

### 3.2. Measuring and Valuing Outcomes

There have been significant efforts to develop methods to value outcomes for interventions that extend beyond health outcomes. Both within and outside of the health care sector, measures of health such as the EQ-5D, which is used to generate the QALY, may not cover all issues that matter to users or decision-makers. It should not be assumed that improvement in health is the key benefit associated with an intervention to enhance the NE. Research is currently underway [33] to extend the QALY, replacing the EQ-5D HRQoL descriptor with an extended quality of life descriptor, well-being [34]. Ultimately, the decision-maker/s that the economic evaluation is intended to inform need to choose what counts in terms of measuring the benefits of interventions. In maintaining the use of a single common measure, this would facilitate the adoption of a broader perspective, reflecting the objectives of decision-maker/s, potentially across sectors.

### 3.3. Cross-Sector Effects

Just as health benefits may not be sufficient to capture the full benefits associated with interventions to enhance the NE, for example, resource use and costs associated with the intervention may also extend beyond one sector. Capturing the full cost impact can be practically challenging, especially where markets do not exist. In addition, there is the issue of who pays for NE interventions, when costs and cost savings fall on multiple budgets across sectors (and indeed who conducts the evaluation itself, which would ideally be undertaken with input from across affected sectors). In practice, shared budgets, such as those observed in Manchester for health and social care [35], may offer the way forward; however, this will require careful consideration where outcomes of interest cannot be measured using common units, such as health or well-being.

Such issues, more broadly, may be better addressed by taking a general equilibrium approach, where a wide range of cross-sector costs and outcomes are relevant. Typically, economic evaluation uses a partial equilibrium approach, which considers the impacts of an intervention in markets that are directly affected. It is assumed that an intervention is assessed according to an existing set of prices and resource use in the broader economy.

### 3.4. Equity Considerations

There have been many efforts to understand the equity impacts of interventions on health and well-being [36,37] and, increasingly, there have been efforts to incorporate consideration of the distributional impact of interventions, that is, directly incorporating health inequality impacts into cost effectiveness analysis [29]. This approach has not been applied in the context of NE interventions to date, however, the general approach should be applicable. What does require further research is how equity impacts across different sectors can be both aggregated and potentially traded off against each other. Where there are multiple outcomes, that is, not just health or well-being, it is also relevant to consider inequalities in all of these outcomes and the eventual distribution of these outcomes across a population.

## 4. Discussions

The evaluation of NE interventions can be difficult and there are multiple challenges, both practical and theoretical. The methodological considerations raised in the paper relate to technologies in the health, as well as the NE, sector and, as seen in the examples provided, the considerations raised are not unique to one particular sector, but rather have relevance across sectors and across technologies. Many of these are not specific to NE evaluations, but are more general issues relating to the evaluation of public health interventions and policies. Recently, there have been significant efforts to consider what makes the evaluation of public health interventions and policies distinct from pharmaceuticals, as an example. These efforts have resulted in an increase in public health evaluation, including economic evaluation, and there have been efforts to invest in research to improve methodology and data collection; however, there are still gaps in knowledge [38].

This increased emphasis has implications across the range of public health interventions and policies, including those pertaining to the natural environment. There are still a number of areas where research is required, including consideration of commonalities and differences across the range of public health contexts, that is, environmental, health promotion, screening, and so on. In particular, the cross-cutting nature of public health, and indeed the NE [39], requires that policies and interventions may need to be assessed, funded, and implemented by various sectors. Where multiple sectors may incur costs or benefits (positive or negative), this raises important questions about who will pay for the interventions relative to who will benefit from any effects. Further methods research is required to formally incorporate the full range of these impacts, whether on a sector by sector basis, or aggregated into a multi-sector analysis [40,41]. Further exploration of what constitutes the appropriate perspective beyond a single sector such as health is required and, in practical terms, this would be facilitated by significant changes in system funding and organization [42]. The best way to make these methodological advances and organisational changes remains an ongoing challenge.

## References

[1] Stafinski T., Menon D., Philippon D.J., McCabe C. (2011). Health technology funding decision-making processes around the world. Pharmacoeconomics.

[2] Sculpher M.J., Claxton K., Drummond M., McCabe C. (2006). Whither trial-based economic evaluation for health care decision making?. Health Econ..

[3] International Society for Pharmacoeconomics and Outcomes Research. https://www.ispor.org/HTADirectory/Index.aspx.

[4] Craig P., Dieppe P., Macintyre S., Nazareth I., Petticrew M. (2008). Developing and evaluating complex interventions: The new MRC guidance. BMJ.

[5] Ramsey S., Willke R., Glick H. (2015). Cost-effectiveness analysis alongside clinical trials II—An ISPOR good research practices task force report. Value Health.

[6] Philips Z., Bojke L., Sculpher M., Claxton K., Golder S. (2006). Good practice guidelines for decision-analytic modelling in health technology assessment: A review and consolidation of quality assessment. Pharmacoeconomics.

[7] Weatherly H., Drummond M., Claxton K., Cookson R., Ferguson B., Godfrey C., Rice N., Sculpher M., Sowden A. (2009). Methods for assessing the cost-effectiveness of public health interventions: Key challenges and recommendations. Health Policy.

[8] Chalmers T.C., Smith H., Blackburn B., Silverman B., Schroeder B., Reitman D., Ambroz A. (1981). A method for assessing the quality of a randomized control trial. Control. Clin. Trials.

[9] Bird E.L., Ige J.O., Pilkington A.P., Burgess-Allen J. (2018). Built and natural environment planning principles for promoting health: An umbrella review. BMC Public Health.

[10] Hunter R.F., Christian H., Veitch J., Astell-Burt T., Hipp J.A., Schipperijn J. (2015). The impact of interventions to promote physical activity in urban green space: A systematic review and recommendations for future research. Soc. Sci. Med..

[11] Humphreys D.K., Panter J., Sahlqvist S., Goodman A., Ogilvie D. (2016). Changing the environment to improve population health: A framework for considering exposure in natural experimental studies. J. Epidemiol. Community Health.

[12] European Commission (2018). Mapping and Assessment of Ecosystems and their Services.

[13] Abhijitha K.V., Kumarab P., Gallagher J., McNabola A., Baldau R., Pilla F., BrianBroderick B., Di Sabatinoh S., Pulvirentii B. (2017). Air pollution abatement performances of green infrastructure in open road and built-up street canyon environments—A review. Atmos. Environ..

[14] Narayan S., Beck M.W., Wilson P., Thomas C.J., Guerrero A., Shepard C.C., Reguero B.G., Franco G., Jn C.I., Trespalacios D. (2017). The value of coastal wetlands for flood damage reduction in the northeastern USA. Sci. Rep..

[15] Fisher B., Costanza R., Turner K.R., Morling P. (2001). Defining and Classifying Ecosystem Services for Decision Making.

[16] Daniels M.J., Dominici F., Samet J.M., Zeger S.L. (2000). Estimating particulate matter-mortality dose-response curves and threshold levels: An analysis of daily time-series for the 20 largest US cities. Am. J. Epidemiol..

[17] Schmitt L.H., Graham H.M., White P.C. (2016). Economic evaluations of the health impacts of weather-related extreme events: A scoping review. Int. J. Environ. Res. Public Health.

[18] Saulnier D.D., Hanson C., Ir P., Molsted Alvesson H., von Schreeb J. (2018). The effect of seasonal floods on health: Analysis of six years of national health data and flood maps. Int. J. Environ. Res. Public Health.

[19] Fernandez A., Black J., Jones M., Wilson L., Salvador-Carulla L., Astell-Burt T., Clack D. (2015). Flooding and mental health: A systematic mapping review. PLoS ONE.

[20] Bowler D.E., Buyung-Ali L., Knight T.M., Pullin A.S. (2010). Urban greening to cool towns and cities: A systematic review of the empirical evidence. Landsc. Urban Plan..

[21] Moller I., Kudella M., Rupprecht F., Spencer T., Paul M., van Wesenbeeck B.K., Wolters G., Jensen K., Bouma T.J., Miranda-Lange M. (2004). Wave Attenuation over Coastal Salt Marshes under Storm Surge Conditions.

[22] (2016). Stern Review: The Economics of Climate Change. http://mudancasclimaticas.cptec.inpe.br/~rmclima/pdfs/destaques/sternreview_report_complete.pdf.

[23] National Institute for Health and Care Excellence (2013). Guide to the Methods of Technology Appraisal 2013.

[24] McCabe C., Claxton K., Culyer A.J. (2008). The NICE cost-effectiveness threshold: What it is and what that means. Pharmacoeconomics.

[25] O’Brien B. (2014). Cost-Benefit Analysis, Willingness to Pay. Statistics Reference Online.

[26] Council of Europe (1950). European Convention on Human Rights.

[27] Dietz S., Atkinson G. (2010). The Equity-Efficiency Trade-off in Environmental Policy: Evidence from Stated Preferences. Land Econ..

[28] Chee Keng Lee A., Jordan H.C., Horsley J. (2015). Value of urban green spaces in promoting healthy living and wellbeing: Prospects for planning. Risk Manag. Healthc. Policy.

[29] Cookson R., Mirelman A., FGriffin S., Asaria M., Dawkins B., Norheim O.F., Verguet S., Culyer A.J. (2017). Using cost-effectiveness analysis to address health equity concerns. Value Health.

[30] World Health Organisation (2016). Urban Green Spaces and Health—A Review of Evidence.

[31] Craig P., Cooper C., Gunnell D., Haw S., Lawson K., Macintyre S., Ogilvie D., Petticrew M., Reeves B., Sutton M. (2012). Using natural experiments to evaluate population health interventions. J. Epidemiol. Community Health.

[32] Public Health England (2018). Estimation of Costs to the NHS and Social Care Due to the Health Impacts of Air Pollution.

[33] Brazier J., Tsuchiya A. (2015). Improving Cross-Sector Comparisons: Going Beyond the Health-Related QALY. Appl. Health Econ. Health Policy.

[34] Brazier J., Peasgood T., Mukuria C., Carlton J., Rowen D., Tsuchiya A., Hernandez M., Van Hout B., Connell J., Johnson J. Extending the QALY. https://scharr.dept.shef.ac.uk/e-qaly/about-the-project/.

[35] Greater Manchester Combined Authority (2015). Taking Charge of Our Health and Social Care in Greater Manchester.

[36] Pickett K.E., Wilkinson R.G. (2015). Income inequality and health: A causal review. Soc. Sci. Med..

[37] Wilkinson R.G., Pickett K. (2016). The enemy between us: The psychological and social costs of inequality. Eur. J. Soc. Psychol..

[38] Smith R.D., Petticrew M. (2010). Public health evaluation in the twenty-first century: Time to see the wood as well as the trees. J. Public Health.

[39] Hutton G. (2000). Considerations in Evaluating the Cost-Effectiveness of Environmental Health Interventions.

[40] Griffin S., Walker S., Sculpher M., Asaria M. (2018). An Analytical Framework for Economic Evaluation of Interventions with Effects on Multiple Outcomes, Costs Falling on Different Budgets, and Involving More than One Decision Maker. J. Int. Soc. Pharmacoecon. Outcomes Res..

[41] Walker S., Griffin S., Claxton K., Palmer S., Sculpher M. (2013). Appropriate Perspectives for Health Care Decisions.

[42] Claxton K., Walker S. (2010). Appropriate Perspectives for Health Care Decisions.

